# m6A and miRNA jointly regulate the development of breast muscles in duck embryonic stages

**DOI:** 10.3389/fvets.2022.933850

**Published:** 2022-10-24

**Authors:** Lihong Gu, Shunjin Zhang, Boling Li, Qicheng Jiang, Tieshan Xu, Yongzhen Huang, Dajie Lin, Manping Xing, Lili Huang, Xinli Zheng, Feng Wang, Zhe Chao, Weiping Sun

**Affiliations:** ^1^Tropical Crops Genetic Resources Institute, Chinese Academy of Tropical Agricultural Sciences, Haikou, China; ^2^Institute of Animal Science and Veterinary Medicine, Hainan Academy of Agricultural Sciences, Haikou, China; ^3^College of Animal Science and Technology, Northwest A&F University, Xianyang, China; ^4^The Hainan Animal Husbandry Technology Promotion Station, Haikou, China; ^5^School of Life Science, Hainan University, Haikou, China; ^6^Key Laboratory of Tropical Animal Breeding and Disease Research, Haikou, China

**Keywords:** ducks, embryo, breast muscles, m6A sequencing, miRNAs sequencing

## Abstract

N6-methyladenosine (m6A) is an abundant internal mRNA modification and plays a crucial regulatory role in animal growth and development. In recent years, m6A modification has been found to play a key role in skeletal muscles. However, whether m6A modification contributes to embryonic breast muscle development of Pekin ducks has not been explored. To explore the role of m6A in embryonic breast muscle development of ducks, we performed m6A sequencing and miRNA sequencing for the breast muscle of duck embryos on the 19th (E19) and 27th (E27) days. A total of 12,717 m6A peaks were identified at E19, representing a total of 7,438 gene transcripts. A total of 14,703 m6A peaks were identified, which overlapped with the transcripts of 7,753 genes at E27. Comparing E19 and E27, we identified 2,347 differential m6A peaks, which overlapped with 1,605 m6A-modified genes (MMGs). Gene Ontology (GO) and Kyoto Encyclopedia of Genes and Genomes (KEGG) analyses revealed that MMGs were enriched in multiple muscle- or fat-related pathways, which was also revealed from our analysis of differentially expressed genes (DEGs). Conjoint analysis of m6A-seq and RNA-seq data showed that pathways related to β-oxidation of fatty acids and skeletal muscle development were significantly enriched, suggesting that m6A modification is involved in the regulation of fat deposition and skeletal muscle development. There were 90 upregulated and 102 downregulated miRNAs identified between the E19 and E27 stages. Through overlapping analysis of genes shared by MMGs and DEGs and the targets of differentially expressed miRNAs (DEMs), we identified six m6A-mRNA-regulated miRNAs. Finally, we found that m6A modification can regulate fat deposition and skeletal muscle development. In conclusion, our results suggest that m6A modification is a key regulator for embryonic breast muscle development and fat deposition of ducks by affecting expressions of mRNAs and miRNAs. This is the first study to comprehensively characterize the m6A patterns in the duck transcriptome. These data provide a solid basis for future work aimed at determining the potential functional roles of m6A modification in adipose deposition and muscle growth.

## Introduction

More than 150 chemical modifications to RNA have been described (1), and these structural modifications play regulatory roles by affecting gene expression. In recent years, with the identification of enzymes capable of reversing N6-methyladenosine (m6A) and the development of transcriptome-wide sequencing methods to map modified sites (2–5), the prevalence and functional significance of internal mRNA modifications have been recognized. Therefore, there is a renewed interest in the biological function of RNA modification.

N6-methyladenosine was first discovered in 1974 and refers to the RNA methylation modification on the sixth N atom of base A with the active adenosine acid as the methyl donor (6). m6A is the most abundant internal mRNA modification and has been observed in various species, accounting for over 80% of all RNA base methylations (7–9). The enzymes that modify m6A methylation include “writers,” “erasers,” and “readers,” which refer to methylated transferases, demethylases, and methylated reading proteins, respectively (10). Methyltransferase-like 3 (METTL3) and methyltransferase-like 14 (METTL14), regulated by the association of a subunit protein Wilms tumor 1-associated protein (WTAP), belong to methylated transferase and can form complexes to catalyze the deposition of m6A in mammalian mRNA (11, 12). AlkB homolog 5 (ALKBH5) and fat mass and obesity-associated (FTO) protein act as m6A demethylases to remove methyl from target regions (13, 14), while heterogeneous nuclear ribonucleoprotein and YTH domain-containing RNA-binding protein act as m6A-methylated reading proteins (15).

N6-methyladenosine is closely involved in the regulation of gene expression, RNA transcription, translation, shearing, degradation, and nuclear transportation (16, 17). More importantly, m6A methylation modification plays an essential role in animal growth and development. METTL3 knockdown inhibited myoblast proliferation and myogenic differentiation, whereas METTL3 overexpression promoted these processes (18). A complete transcriptome map of m6A was obtained by transcriptome sequencing of muscle tissue from three different pig breeds, and m6A was found to be widely distributed in muscle tissue (19). However, to our knowledge, no study has addressed m6A modification in the breast muscle tissues of ducks.

China is a major producer and consumer of ducks in the world, and in 2019, duck production and consumption in China accounted for about 75% of the world's duck stock, according to the Food and Agriculture Organization (FAO). Pekin duck is a famous meat breed for its fast growth rate. Therefore, a study on the regulation mechanism of breast muscle development is crucial for improving the meat yield of Pekin duck and is also the basis of breeding a new lean meat type of Pekin duck. Our previous research showed that the 19th day of hatching (E19) is the fastest point of breast muscle development—as well as the crucial transition point for breast muscle development during the embryonic stage of Pekin ducks—and that the weight of the breast muscles was largely constant from E19 to E27 (20). Subsequently, miRNA and mRNA patterns of breast muscles at the E13 (13th day of hatching), E19, and E27 (27th day of hatching) stages were studied (21, 22), and candidates that may play key roles in the breast muscle development of Pekin duck were identified. However, no studies have shown whether m6A and miRNA expressions are different in E19 and 27 and the potential role of m6A in the breast muscle development of Pekin duck.

The aim of this study was to explore whether m6A and miRNA cooperatively regulate breast muscle differentiation in Pekin duck embryos. We conducted m6A sequencing on the breast muscle tissues of ducks at E19 and E27 to explore whether m6A modification existed in these two periods. We also performed miRNA-seq to explore whether some genes were regulated by both m6A and miRNA in Pekin duck embryos. We determined the distribution of m6A and miRNA regulation during breast muscle differentiation in Pekin duck embryos. The results of this study will offer a basis for unraveling the role of m6A modification and miRNA in breast muscle differentiation.

## Materials and methods

### Ethics approval

This study was approved by the Institute of Animal Science & Veterinary Medicine, Hainan Academy of Agricultural Sciences (IASVM-HAAS, Haikou, China; ethical approval reference number: IASVMHAAS-AE-202012), and followed the Regulations for the Administration of Affairs Concerning Experimental Animals of China.

### Searching of duck homologous sequences in m6A RNA modification

The duck reference genome sequences (BGI_duck_1.0) and complete genome annotation GFF3 file were downloaded from the NCBI database, which ensured that genes affected by m6A methylation were accurately located. Related enzyme sequences in m6A RNA modification were searched by blasting the duck reference genome, and the duck sequences were compared with those of human.

### Sample collection

The experimental duck embryos were obtained from the Z-type Pekin Duck Breeding Farm of the Beijing Institute of Animal Science, Chinese Academy of Agricultural Sciences. The eggs were incubated at a temperature of 37 ± 0.5°C and a humidity of 86–87%. The breast muscle samples were obtained from E13, E19, and E27 stages. The duck embryo and breast muscle were taken out and spun off under aseptic conditions. Three eggs at each stage were used to collect breast muscles. The left and right breast muscles were placed in separate centrifuge tubes, placed in liquid nitrogen, taken back to the laboratory, and stored in a −80°C cryogenic refrigerator. The left and right breast muscles from each duck embryo were used for m6A-seq and miRNA-seq sequencing analyses, respectively.

### RNA extraction

Total RNA was isolated and purified using Trizol reagent (Invitrogen, Carlsbad, CA, USA) following the manufacturer's instructions. The RNA concentration and purity of each sample were quantified using a NanoDrop ND-1000 (NanoDrop, Wilmington, DE, USA). The RNA integrity was assessed by a Bioanalyzer 2100 (Agilent, CA, USA) with RIN number >7.0 and confirmed by electrophoresis with denaturing agarose gel. Poly (A) RNA was purified from 50 μg total RNA using Dynabeads Oligo (dT)25-61005 (Thermo Fisher, CA, USA). Then, the poly (A) RNA was fragmented into small pieces using a Magnesium RNA Fragmentation Module (NEB, cat. e6150, USA) at 86°C for 7 min.

### Expression pattern of m6A-associated methylase

Total RNA of each breast muscle sample from three embryonic stages was isolated using Trizol reagent (Invitrogen, USA) following the manufacturer's instructions. The SYBR PrimeScript RT-PCR Kit (TaKaRa, Japan) was used for reverse transcription polymerase chain reaction (RT-PCR). The relative expression levels of *METTL3, METTL14, WATP, KIAA1429, FTO, ALKBH5, YTHDF1*, and *YTHDF2* were examined by quantitative RT-PCR (qRT-PCR) using reference gene GAPDH. The primer sequences are listed in Table 1.

**Table 1:** Primers information of genes.

**Primer labels**	**Sequence (5^′^−3^′^)**	**Annealing temperature °C**
GAPDH-F	CACACGAAGACAGTGGATG	60
GAPDH-R	GAGGCTGGGGATAATGTTCTG	
METTL3-F	GCTCCACCAGCCATAAACC	56
METTL3-R	TGAACTGCGCCACCACAT	
METTL14-F	TGAACAGTAAGGATGACCA	60
METTL14-R	TTGGAGCAGAGGTATCATAA	
WATP-F	TCCAGGAGAATCAAGAGC	53
WATP-R	CATTGCTTGGTCCGTTAG	
KIAA1429-F	TTCTTCTTGCCAGCCTATG	55
KIAA1429-R	ATCCCAGTGTATCCGAGTA'	
FTO-F	ACCTGCTGAAGAAACTTATGAT	60
FTO-R	TTGGTGAAGTGGTATTGCTAAT	
ALKBH5-F	GGAGGGTTACACCTACGGC	57
ALKBH5-R	CCTGATGGGTTTGAACTGGA	
YTHDF1-F	GACTCAACCACAGTATCAGA	60
YTHDF1-R	GTTACCAGTTCCTCCACTT	
YTHDF2-F	CTCTCACGGCTTCCTAAT	60
YTHDF2-R	CGCTTCTGTTGGTCTTATC	

Quantitative RT-PCR was carried out with an iCycler IQ5 Multicolor Real-Time PCR Detection System (Bio-Rad, USA). The qRT-PCR contained 1 μL of cDNA, 12.5 μL of SYBR Premix Ex-Taq, 10.5 μL of ddH_2_O, and 0.5 μL of 10 pmol/μL forward and reverse primer (Table 1). The thermal cycling parameters were one cycle at 95°C for 30 s and 40 cycles at 95°C for 10 s and 60°C for 40 s. An 80-cycle melting curve analysis was performed after each PCR run to confirm product specificity, with one cycle at 95°C for 1 min, one cycle at 55°C, and then increasing temperature of 0.5°C for every 10 s until 95°C while fluorescence was continuously monitored. qRT-PCR analysis of each sample was repeated three times.

### m6A immunoprecipitation (IP), library construction, and sequencing

The cleaved RNA fragments were incubated at 4°C for 2 h with an m6A-specific antibody (Synaptic Systems, cat. 202003, Germany) in IP buffer (50 mM Tris–HCl, 750 mM NaCl, and 0.5% Igepal CA-630). Then, the IP RNA was reverse transcribed to create the cDNA by SuperScript™ II reverse transcriptase (Invitrogen, cat. 1896649, USA), which was then used to synthesize U-labeled second-stranded DNAs with *E. coli* DNA polymerase I (NEB, cat. m0209, USA), RNase H (NEB, cat. m0297, USA), and dUTP solution (Thermo Fisher, cat. R0133, USA). An A-base was then added to the blunt ends of each strand for ligating to the indexed adapters. Each adapter contained a T-base overhang for ligating the adapter to the A-tailed fragmented DNA. Single- or dual-index adapters were ligated to the fragments, and the sample size selection was performed with AMPureXP beads. After the heat-labile UDG enzyme (NEB, cat. m0280, USA) treatment of the U-labeled second-stranded DNAs, the ligated products were amplified by PCR under the following conditions: denaturation at 95°C for 3 min, eight cycles of denaturation at 98°C for 15 s, annealing at 60°C for 15 s, extension at 72°C for 30 s, and final extension at 72°C for 5 min. The average insert size for the final cDNA library was 300 ± 50 bp. Finally, the 2 × 150bp paired-end sequencing (PE150) was performed on an Illumina NovaSeq™ 6000 (LC-Bio Technology Co., Ltd., Hangzhou, China) following the vendor's recommended protocol.

### Data analysis of m6A-seq and RNA-seq

The fastp tool (https://github.com/OpenGene/fastp) was used to remove the reads that contained adaptor contamination, low-quality bases, and undetermined bases with default parameters. Then, sequence quality of IP and Input samples was also verified using fastp. We used HISAT2 (http://daehwankimlab.github.io/hisat2) (23) to map reads to the reference genome of *Anas platyrhynchos*. The mapped reads of IP and Input libraries were provided to an R package exomePeak (https://bioconductor.org/packages/exomePeak), which can identify m6A peaks with bed or bigwig format files that can be adapted for visualization on the IGV software (http://www.igv.org). Peaks were examined by the Poisson distribution matrix with default parameters (*P* < 0.05). MEME (http://meme-suite.org) and HOMER (http://homer.ucsd.edu/homer/motif) were used for *de novo* and known motif findings, followed by localization of the motif with respect to peak summit. Called peaks were annotated by intersection with gene architecture using the R package ChIPseeker (https://bioconductor.org/packages/ChIPseeker). Then, StringTie (https://ccb.jhu.edu/software/stringtie) was used to determine the expression level for all mRNAs from Input libraries by calculating FPKM [total exon fragments/mapped reads (millions) × exon length (kB)]. The differentially expressed genes (DEGs) were selected with log2 (fold change) >1 or log2 (fold change) < -1 and *p-*value < 0.05 using the R package edgeR (https://bioconductor.org/packages/edgeR) (24). Gene enrichment analysis was performed by Gene Ontology (GO) (http://www.geneontology.org/) and Kyoto Encyclopedia of Genes and Genomes (KEGG) (http://www.kegg.jp/).

### Conjoint analysis of m6A-seq and RNA-seq data

To comprehensively study the roles of methylated m6A level and gene expression abundance, we performed correlation analyses of m6A-seq and RNA-seq data. Through the analyses of m6A-seq and RNA-seq data, we obtained differentially methylated m6A peaks in abundance and DEGs. We divided the differentially methylated m6A peaks into upregulated m6A sites (higher methylated m6A sites at E27 than those at E19) and downregulated m6A sites (higher methylated m6A sites at E19 than those at E27). Similarly, DEGs were also divided into upregulated genes (higher expression levels at E27 than those at E19) and downregulated genes (higher expression levels at E19 than those at E27). We overlapped up- and down-methylated m6A sites with up- and downregulated genes and then obtained the upregulated genes with upregulated methylated m6A sites (up–up), downregulated genes with upregulated methylated m6A sites (down–up), upregulated genes with downregulated methylated m6A sites (up–down), and downregulated genes with hypo-methylated m6A sites (down–down). GO and KEGG functional analyses for genes shared by DEGs and DMGs were performed to investigate the functions of these genes.

### miRNA library construction, sequencing, and data analysis

The miRNA libraries were constructed with a similar method to Gu et al. (22). In brief, the isolated total RNA of each individual from E19 and E27 was used for the generation of the small RNA libraries where the population of recovered small RNAs, ranging in size from 18 to 30 nucleotides, was purified using 15% polyacrylamide gel. Then, 5**′** adaptors (Illumina, USA) were ligated to the purified small RNAs, followed by purification of ligation products on Novex 15% TBE–urea gel. The 5**′** ligation products were then ligated to 3**′** adaptors (Illumina), and products with 5**′** and 3**′** adaptors were purified using Novex 10% TBE–urea gel (Invitrogen). Subsequently, reverse transcription reactions were performed using the RT primer, and PCRs were performed using the forward and reverse Illumina primers. The PCR product was purified by phenol/chloroform extraction and ethanol precipitation, and miRNA libraries were obtained. After purification and quality detection, miRNA libraries were sequenced on an Illumina Genome Analyzer (LC-Bio Technology Co., Ltd., Hangzhou, China), and raw reads were produced.

The raw reads were subjected to an in-house program, ACGT101-miR (LC Sciences, Houston, TX, USA), to remove adapter dimers, junk, low complexity, common RNA families (rRNA, tRNA, snRNA, and snoRNA), and repeats. Subsequently, unique sequences with lengths of 18–26 nucleotides were mapped to specific species precursors in miRBase 22.0 by BLAST search to identify known miRNAs and novel 3p- and 5p-derived miRNAs. Length variation at both the 3**′** and 5**′** ends and only one mismatch inside of the sequence were allowed in the alignment. The unique sequences mapping to specific species mature miRNAs in hairpin arms were identified as known miRNAs. The unique sequences mapping to the other arm of known specific species precursor hairpin opposite to the annotated mature miRNA-containing arm were considered as novel 5p- or 3p-derived miRNA candidates.

The unmapped sequences were BLASTed against the specific genomes, and the hairpin RNA structures containing sequences were predicated from the flank 80 nt sequences using RNAfold software (http://rna.tbi.univie.ac.at//cgi-bin/RNAWebSuite/RNAfold.cgi). The criteria for secondary structure prediction were as follows: (1) number of nucleotides in one bulge in stem (≤ 12); (2) number of base pairs in the stem region of the predicted hairpin (≥16); (3) cutoff of free energy (kCal/mol ≤ -15); (4) length of the hairpin (up and down stems + terminal loop ≥50); (5) length of the hairpin loop (≤ 20); (6) number of nucleotides in one bulge in the mature region (≤ 8); (7) number of biased errors in one bulge in the mature region (≤ 4); (8) number of biased bulges in the mature region (≤ 2); (9) number of errors in the mature region (≤ 7); (10) number of base pairs in the mature region of the predicted hairpin (≥12); and (11) percent of mature in stem (≥80).

Differentially expressed miRNAs were selected by the t-test method (http://en.wikipedia.org/wiki/Students_t-test), which compares the significance level of difference between the E19 and E27 stages. A heatmap was used to analyze the cluster pattern in different control sets with log10 values. Target genes of DEMs with a significant difference were predicted by the TargetScan algorithm (25–27) with default parameter and miRanda algorithm (28, 29) (Max_Energy <-10) according to the score standard. Finally, the overlapped genes predicted by both algorithms were deemed as the target genes of DEMs.

### Integrative analysis of miRNA-seq, mRNA-seq, and m6A-seq data

Differentially expressed miRNAs were identified through the analysis of miRNA-seq data, and we predicted the targets of DEMs. Then, we overlapped the targets of DEMs and the genes shared by MMGs and DEGs (called m6A-mRNA-miRNA genes). Subsequently, we identified the miRNAs that were targeted by m6A-mRNA-miRNA genes (called m6A-mRNA-regulated miRNAs). Finally, we performed GO and KEGG functional enrichment analyses of targets of m6A-mRNA-regulated miRNAs to study their potential roles.

### Availability of data

Sequences are available from GenBank with the Bioproject accession numbers SRR13051312–SRR13051329.

## Results

### Comparison of homologous genes of methylase

The sequences of m6A-methylated enzymes in ducks were obtained by blasting on NCBI. Through comparison with human homologous genes of methylases, we found that all duck *METTL14, FTO*, and *YTHDF1* genes had homologous genes in humans. All the E-values of these three genes were 0.0, and their max scores were 674, 605, and 802, respectively.

### m6A modification levels and the expression of m6A RNA modification enzymes

**Figure 1:**
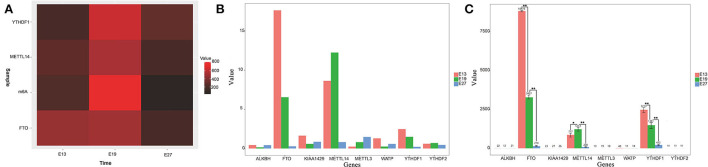
Gene expression levels of m6A methylation enzymes in different stages. **(A)** Heatmap of m6A modification levels of E13, E19, and E27 in duck embryonic breast muscles. **(B)** Gene expression levels of m6A methylation enzymes at E13, E19, and E27 from RNA-seq data. **(C)** Detection of gene expression levels of m6A methylation enzymes at E13, E19, and E27 detected by qRT-PCR. Statistically significant differences are indicated by *p ≤ 0.05, **p ≤ 0.01.

We previously found that duck breast muscles grew much faster at E19 than those at E13 and E27. To explore whether m6A modification played a role in duck embryonic breast muscle development, we examined the levels of m6A in the total RNAs of breast muscles at E13, E19, and E27. Using the colorimetric m6A quantification strategy, we found that the m6A level at E19 was much higher than those at E13 and E27 (Figure 1A), which indicated that the expression of the total RNA methylation level of m6A in duck breast muscle was significantly higher in the period of vigorous proliferation and differentiation than that during the period of cessation of proliferation.

According to our previous transcriptome sequencing (RNA-seq) results, the expression of *METTL14, FTO*, and *YTHDF1* was significantly different among the E13, E19, and E27 stages (*P* < 0.01) (Figure 1B). Then, qRT-PCR was used to test the mRNA expression levels of several m6A RNA modification enzymes during different stages. The results revealed that the expression levels of *METTL14, FTO, YTHDF1*, and *YTHDF2* were higher in the breast at E19 than those at E27, while *WATP, KIAA1429*, and *ALKBH* were more highly expressed at E27 than at E19 (Figure 1C). Therefore, we speculated that the expression of methylase was significantly correlated with the proliferation and differentiation of duck breast muscle cells.

### Transcriptome-wide m6A modification patterns

Through sequencing of m6A-seq and RNA-seq, 68.19–81.52 million clean reads were generated from each m6A-seq dataset and 65.48–88.88 million clean reads were generated from the RNA-seq dataset (Table 2). HISAT2 was used to map reads to the genome of *Anas platyrhynchos* (BGI_duck_1.0) with default parameters. The percentages of mapped reads for m6A-seq ranged from 75.25 to 77.01% and for RNA-seq ranged from 74.45 to 77.62% (Supplementary Table S1).

**Table 2:** Descriptive statistics of sequenced data.

**Sample_ID**	**Raw_Reads**	**Raw_Bases**	**Clean reads**	**Clean bases**	**Clean %**	**Q30%**	**GC%**
E19_1_IP	87574914	13.14G	81524514	11.42G	86.90	93.80	56.63
E19_2_IP	84509772	12.68G	79209738	10.66G	84.07	94.38	55.98
E19_3_IP	74910974	11.24G	70367178	9.77G	86.99	93.59	55.31
E27_1_IP	71935808	10.79G	68192750	9.47G	87.75	94.02	55.20
E27_2_IP	74993630	11.25G	70405880	9.40G	83.54	93.80	54.21
E27_3_IP	75376878	11.31G	70574208	9.60G	84.93	94.30	55.61
E19_1_input	70475228	10.57G	65476148	8.59G	81.23	94.65	57.09
E19_2_input	88583582	13.29G	82522530	10.06G	75.72	94.86	56.84
E19_3_input	70391384	10.56G	66478036	9.16G	86.75	94.40	56.41
E27_1_input	86824864	13.02G	81526132	10.73G	82.36	94.75	55.79
E27_2_input	93174910	13.98G	88875790	11.83G	84.68	94.66	55.35
E27_3_input	94960962	14.24G	88872294	11.39G	79.97	94.54	56.37

**Figure 2:**
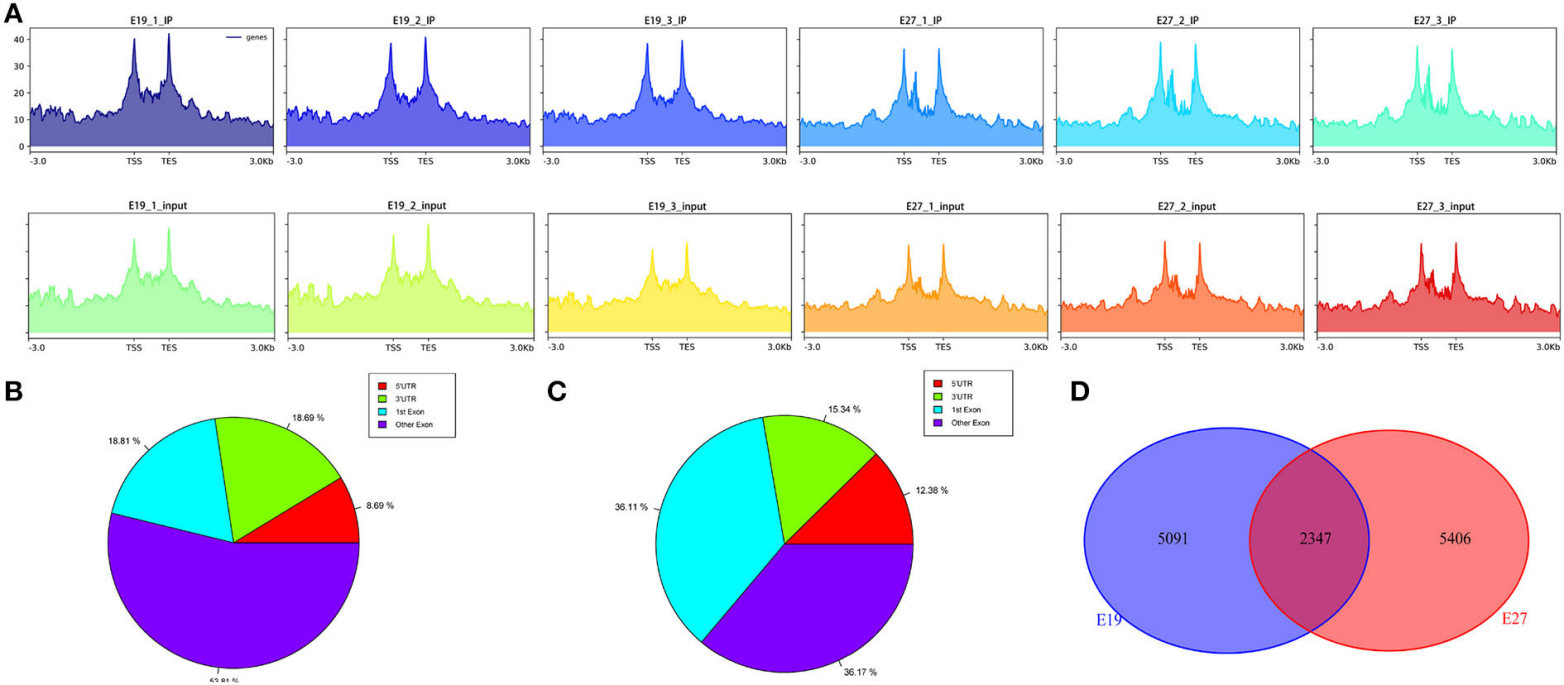
Analysis of transcriptome-wide m6A-seq data. **(A)** Distribution of reads in the upstream and downstream 3kb range from the TSS–TES (transcription end sites). The abscissa is the gene location, and the ordinate is the coverage depth of reads. **(B)** Pie chart of m6A peaks at E19. **(C)** Pie chart of m6A peaks at E27. **(D)** Pie chart of unique and shared m6A peaks between E19 and E27.

To study the distribution of m6A peaks, we first detected the enrichment of reads near the transcriptome initiation site (transcription start sites, TSS). The results showed that the m6A peaks were enriched in the vicinity of transcription start sites (TSS) (Figure 2A). We then divided the duck genes into 5'UTR, 3'UTR, first exon, and other exons and found that the reads from m6A-IP samples are highly accumulated around the other exons (except the first exon) at both E19 (53.81%) and E27 (36.17%) stages (Figures 2B,C). For E19, the ratios of peaks were similar on the first exon (18.81%) and 3**′**UTR (18.69%), while peaks distributed at 5′UTR were the least (8.69%). For E27, the percentages of peaks on the first exon and the other exons were also similar, all accounting for about 36%. A total of 12,717 m6A peaks were identified by exomePeak at E19, representing the transcripts of 7,438 genes. At E27, 14,703 m6A peaks were identified, which correspond to the transcripts of 7,753 genes. There were 5,091 and 5,406 unique peaks for E19 and E27, but only 2,347 peaks were shared by E19 and E27, indicating their significant difference in global m6A modification patterns (Figure 2D).

### Enrichment analysis of m6A-modificated genes

**Figure 3:**
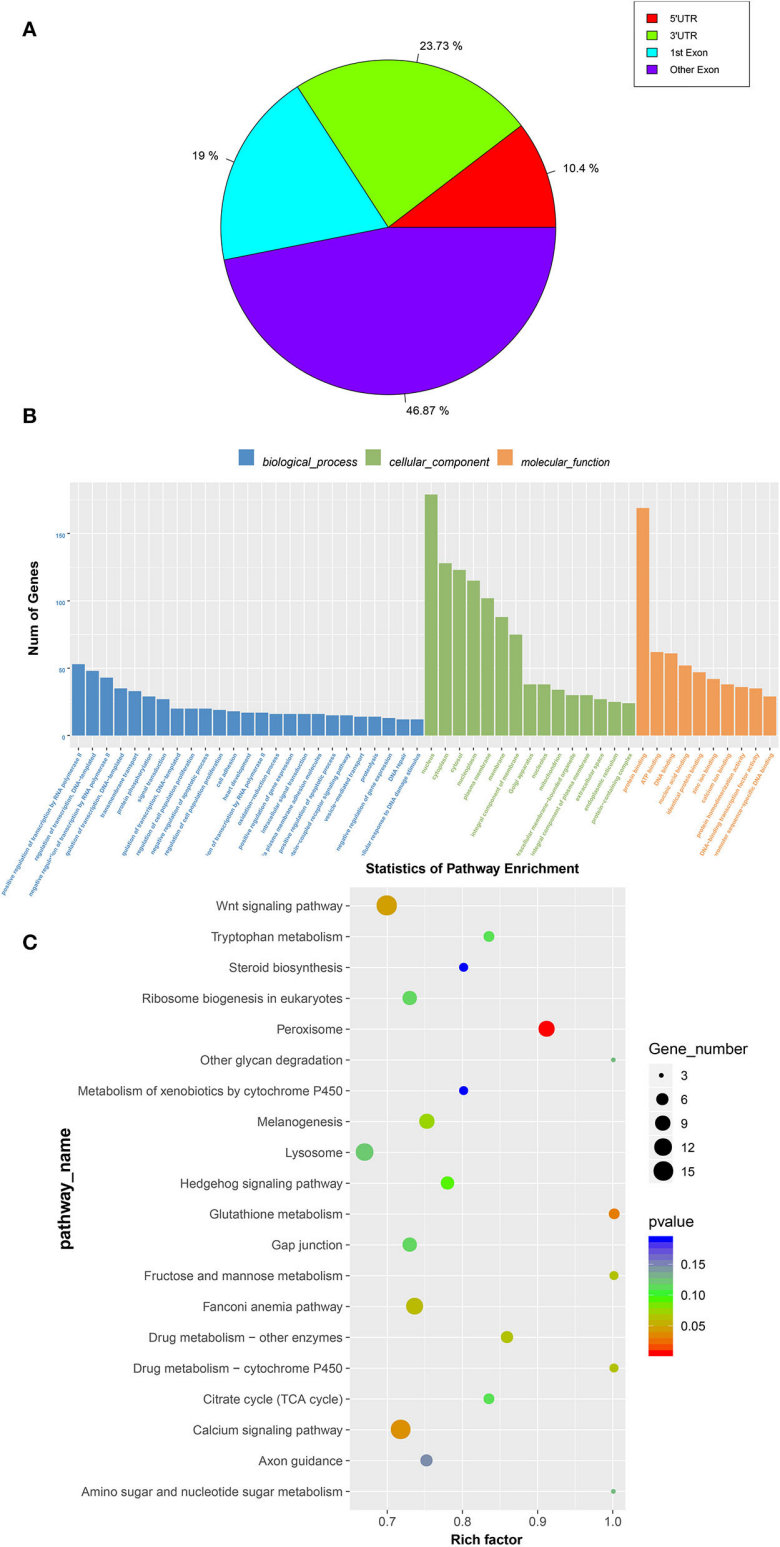
GO and KEGG analyses of m6A-modified genes. **(A)** Distribution of m6A-modified genes (MMGs) along genes. **(B)** Analysis of GO enrichment. **(C)** Statistics of KEGG pathway enrichment.

The abundance of the m6A peaks between the E19 and E27 stages was compared to identify the abundance differential peaks (Supplementary material 1). We found 2,347 differential m6A peaks between the E19 and E27 stages, which overlapped 1,605 genes (called m6A-modified genes, MMGs). The MMGs were mainly located at other exons and 3**′**UTR regions (Figure 3A). GO and KEGG analyses were then performed to explore the function of m6A-modified genes. GO analysis displayed that MMGs were mostly enriched in cellular component and molecular functions (Figure 3B). Moreover, there were 179 genes located in the nucleus. Furthermore, 169 genes had a vital impact on protein binding. Pathway analysis revealed that peroxisome was the most significantly enriched pathway, and m6A-modified genes were significantly enriched in the Wnt signaling pathway and calcium signaling pathway (Figure 3C).

### Identification of differentially expressed genes

**Figure 4:**
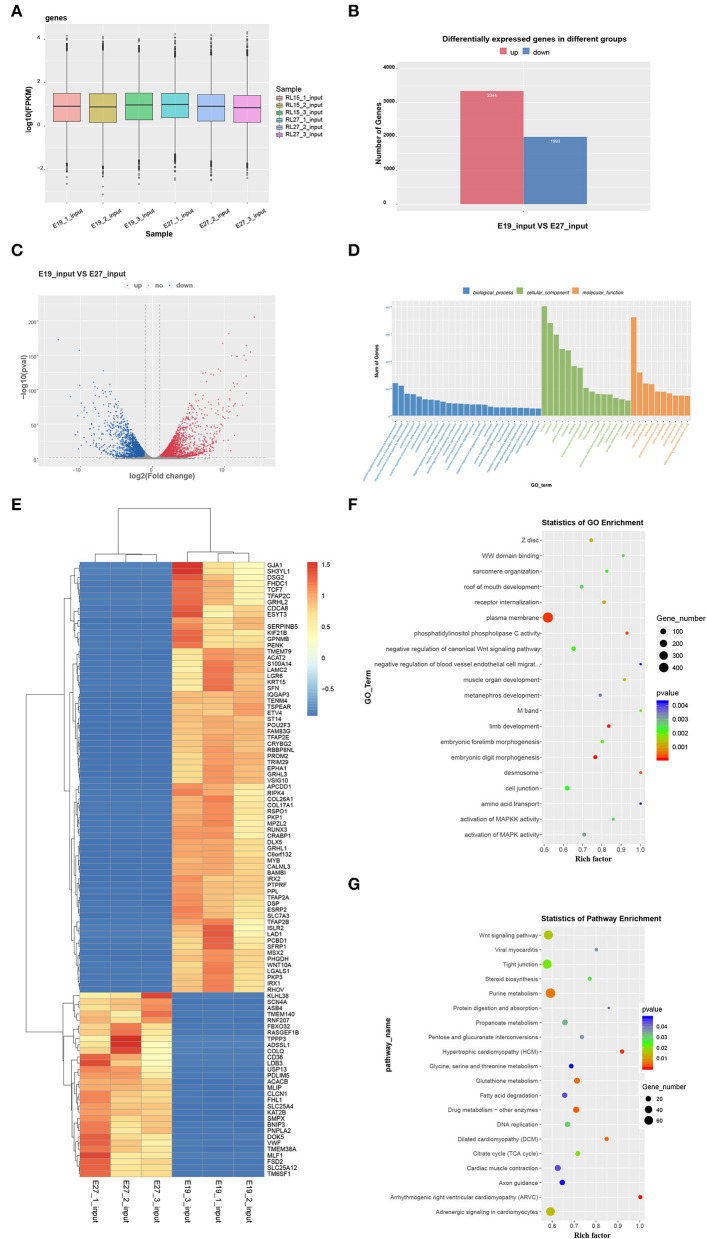
Global gene expression in the two stages and GO and KEGG analyses of DEGs. **(A)** Global expression levels of expressed genes. **(B)** Number of upregulated and downregulated DEGs between the two stages. **(C)** Volcanic map of DEGs. **(D)** Heatmap of DEGs. **(E)** Statistics of GO enrichment. **(F)** GO analysis of DEGs. **(G)** KEGG analysis of DEGs.

There were 12,869 genes detected at E19 and E27. The number of genes expressed in three individuals ranged from 11,226 to 11,494 at E19, while that ranged from 9,966 to 10,491 at E27. In general, the number of expressed genes was higher at E19 than that at E27. However, the overall gene expression levels were similar in both periods, which was shown from the gene expression box plot (Figure 4A). We also found 5,337 DEGs between these two stages, including 3,344 highly expressed genes at E27 and 1,993 highly expressed genes at E19 (Figures 4B–D).

Differentially expressed genes were selected for Gene Ontology (GO) and KEGG pathway enrichment analyses. According to the GO results, DEGs were not directly involved in the biological process, but more concentrated in the aspects of cellular components and molecular function. Eight hundred and three DEGs were enriched in the nucleus, and 681 DEGs were enriched in the cytoplasm. Furthermore, 723 DEGs participated in the molecular function of protein binding (Figure 4E). Through GO enrichment analysis, we found that the plasma membrane was the most significant term with the largest number of genes (436 genes). In addition, the selected DEGs were enriched in some skeletal muscle development and fat deposition GO terms, such as negative regulation of canonical Wnt signaling pathway, muscle organ development, activation of MAPKK activity, and activation of MAPK activity (Figure 4F). Moreover, KEGG analysis showed that DEGs were enriched in several pathways related to muscle development. For example, the Wnt signaling pathway was enriched, which is also related to skeletal muscle development. Additionally, pathways of dilated cardiomyopathy (DCM), fatty acid metabolism, viral myocarditis, and cardiac muscle contraction—which are associated with fat deposition and cardiac muscle development—were revealed through KEGG analysis (Figure 4G).

### Conjoint analysis of m6A-seq and RNA-seq data at E19 and E27

**Figure 5:**
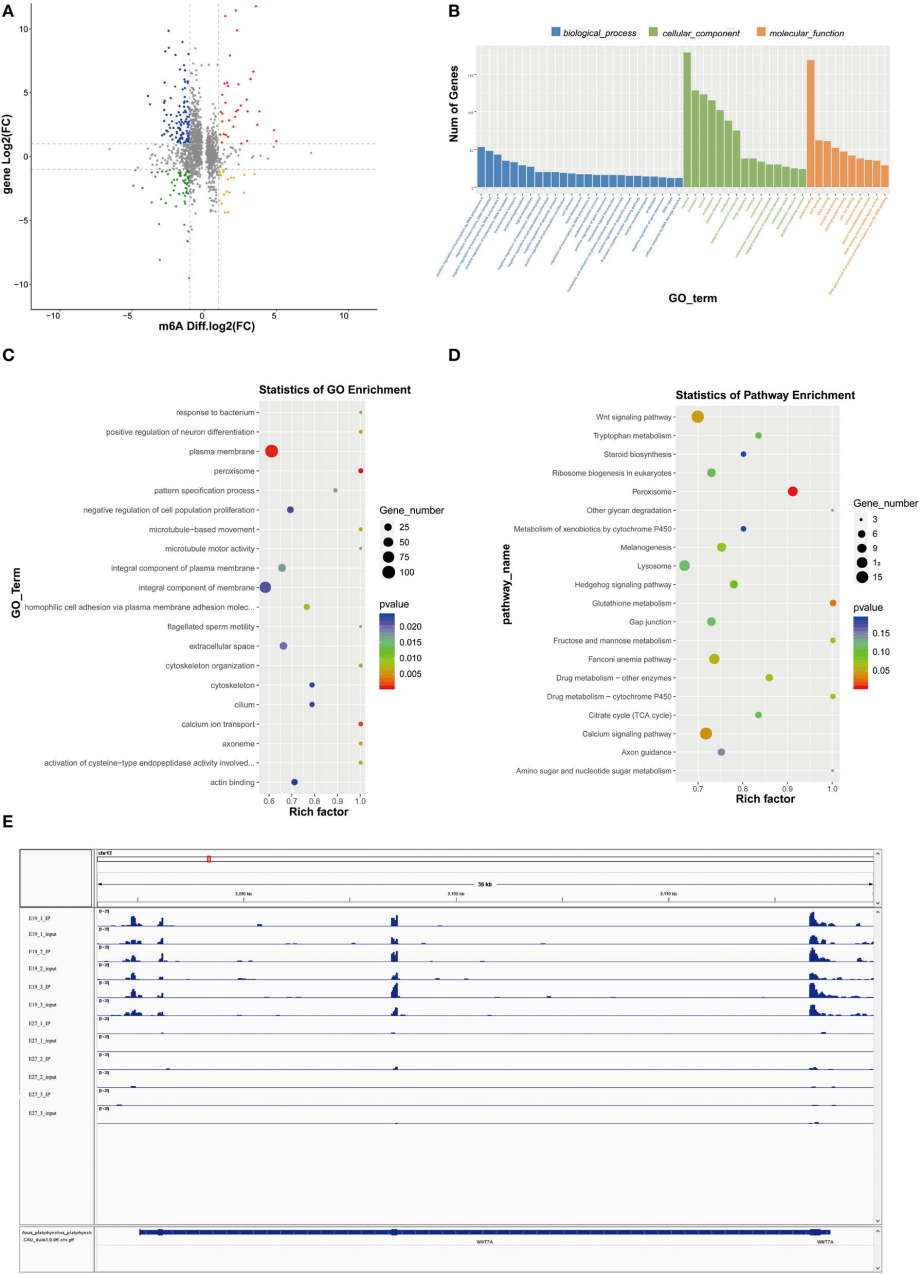
Conjoint analysis of m6A-seq and RNA-seq data. **(A)** Distribution of genes with a significant change in both m6A methylation level and gene expression between E19 and E27. Up–up, up–down represent genes with increased m6A methylation level and increased gene expression and genes with increased m6A methylation level and decreased gene expression, respectively. Down–up, down–down represent genes with decreased m6A methylation level and increased gene expression and genes with decreased m6A methylation level and decreased gene expression, respectively. **(B)** GO terms of genes shared by MMGs and DEGs. **(C)** GO enrichment analysis of genes shared by MMGs and DEGs. **(D)** KEGG enrichment analysis of genes shared by MMGs and DEGs. **(E)** Visualization of m6A abundances in *WINT7a* mRNA transcripts at E19 and E27.

As mentioned above, we had found 2,347 differential m6A peaks between the E19 and E27 stages, with 1,512 downregulated peaks and 835 upregulated peaks in abundance. Through conjoint analysis of m6A-seq and RNA-seq data, the downregulated peaks overlapped with 394 decreased expression genes (down–down genes) and 689 increased expression genes (down–up genes). Conversely, 227 genes with upregulation in m6A abundance showed downregulated gene expression (up–down genes) and 380 genes with upregulation in abundance showed upregulated gene expression (up–up genes) (Figure 5A, Supplementary material 1).

Gene Ontology analysis for genes shared by MMGs and DEGs showed that plasma membrane, peroxisome, and calcium ion transport were the most significant three GO terms (Figures 5B,C), among which peroxisome is involved in β-oxidation of fatty acids and calcium ion transport is involved in muscle development), indicating some genes shared by MMGs and DEGs are potential regulators of skeletal muscle development and fat deposition.

Kyoto Encyclopedia of Genes and Genomes analysis showed that peroxisome (related to β-oxidation of fatty acids), Wnt signaling pathway, and calcium signaling pathway (tightly associated with skeletal muscle development) (Figure 5D) were the most significantly enriched pathways (the peroxisome pathway ranked the first), which were consistent with the GO results described above. In addition, we found many genes related to skeletal muscle development in MMGs such as *BCL9, SOX11, EPHB1, MYOCD, BVES, SLC8A3, ASB2, CFLAR, EPHB1, WNT7A*, and *SCN4A* (Table 3), suggesting that m6A modification plays crucial roles in duck muscle development. Among the skeletal muscle development-related genes, we picked out *Wnt7a* to compare the status of m6A modification levels in various stages and samples using Integrative Genomics Viewer (IGV) software (30). We also found that the m6A levels on the *Wnt7a* gene were significantly different when comparing E19 and E27 (Figure 5E).

**Table 3:** List of 11 genes that exhibit a significant change in both m6A modification and mRNA expression related to muscle development in duck embryonic breast muscle tissues.

**Gene name**	**Chromosome**	**Peak start**	**Peak end**	**Peak length**	**Transcript ID**	**Pattern**	***P*-value**
BCL9	chr1	95237263	95242335	5073	ENSAPLT00000032721	Down–up	1.79414E-11
MYC	chr2	146787275	146787752	478	ENSAPLT00000014574	Down–up	0.000850783
BVES	chr3	71742456	71742814	359	ENSAPLT00000040909	Up–down	9.19716E-45
SOX11	chr3	78759336	78759927	592	ENSAPLT00000046689	Down–up	6.90452E-07
SLC8A3	chr5	28282453	28282632	180	ENSAPLT00000016237	Up–down	5.18455E-16
ASB2	chr5	48558337	48559238	902	ENSAPLT00000011413	Up–down	9.51065E-58
CFLAR	chr7	98022	99193	1172	ENSAPLT00000015969	Up–down	0.045676673
EPHB1	chr9	10474372	10474638	267	ENSAPLT00000034231	Up–down	1.85369E-09
WNT7A	chr13	3117467	3117616	150	ENSAPLT00000014453	Up–down	0.000245641
MYOCD	chr19	620339	620964	626	ENSAPLT00000046004	Up–down	1.04238E-21
SCN4A	chr28	715525	716182	658	ENSAPLT00000005205	Up–down	5.4444E-155

### Association analysis of miRNAs-seq, mRNA-seq, and m6A-seq data

We also tested the differentially expressed miRNAs (DEMs) between the E19 and E27 stages. There were 90 upregulated miRNAs and 102 downregulated miRNAs between the E19 and E27 stages. Through overlapping analysis of genes shared by MMGs and DEGs and the targets of DEMs, we identified six m6A-mRNA-regulated miRNAs, namely, cli-miR-1467-3p_1ss19AG, PC-3p-28816_21, efu-miR-9226_2ss4AG22GA, gga-miR-338-5p, gga-miR-338-3p, and apl-miR-11588-3p.

**Figure 6:**
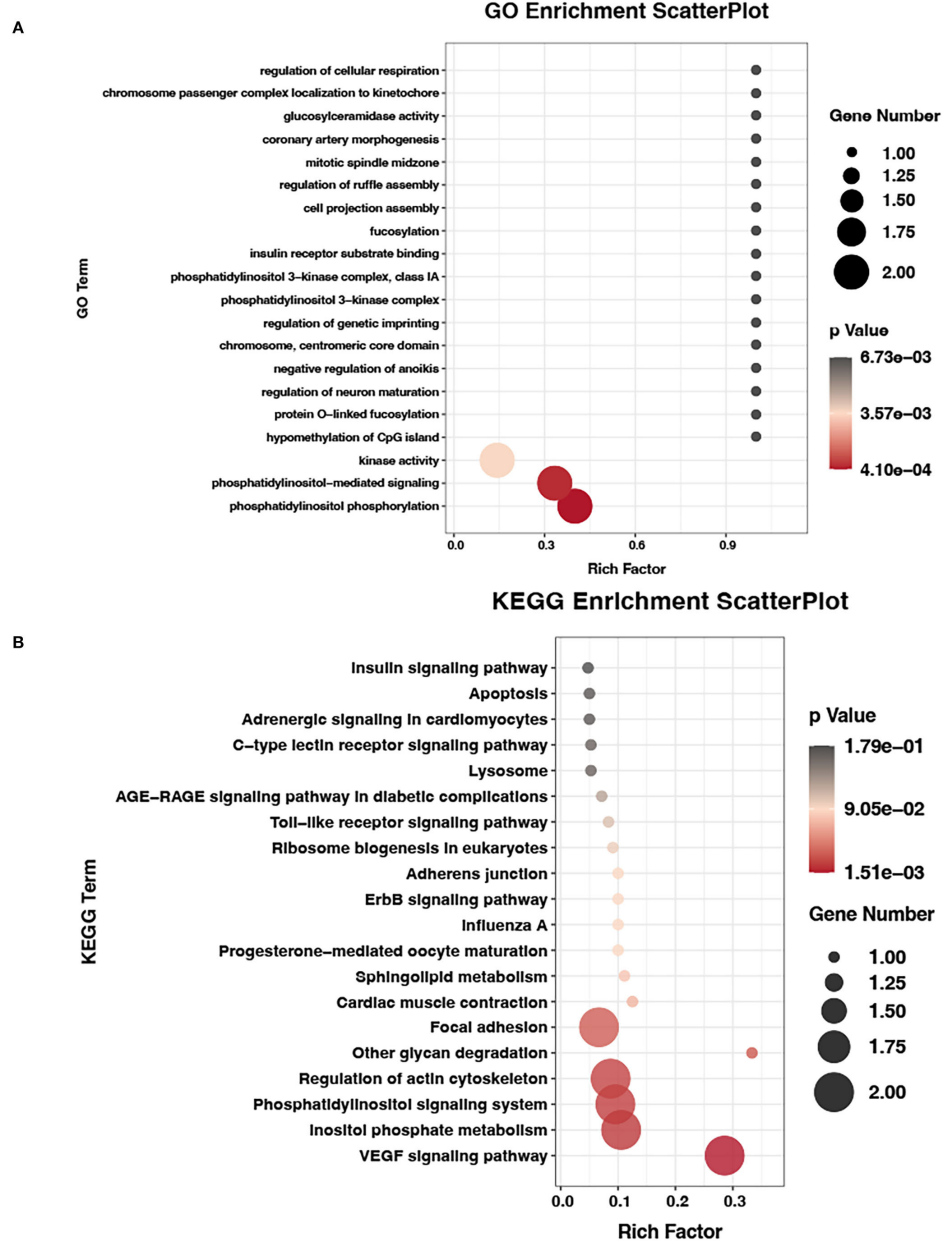
GO and KEGG analyses of m6A-mRNA-regulated miRNAs data. **(A)** GO terms of target genes of m6A-mRNA-regulated miRNAs. **(B)** KEGG enrichment analysis of target genes of m6A-mRNA-regulated miRNAs.

To verify the potential role of m6A-mRNA-regulated miRNAs, we performed the GO and KEGG analyses of the targets of m6A-mRNA-regulated miRNAs. The GO results showed that the most significant GO term (*P* = 4.10E-04) was phosphatidylinositol phosphorylation, which is involved in skeletal muscle development. Then, the second most significant GO term (phosphatidylinositol-mediated signaling) and other significant GO terms (kinase activity, phosphatidylinositol 3-kinase complex, and phosphatidylinositol 3-kinase complex, class IA) were all associated with skeletal muscle development (Figure 6A, Supplementary material 2). Being consistent with the GO results, the KEGG pathway analysis also found that some pathways were involved in skeletal muscle development, such as inositol phosphate metabolism, phosphatidylinositol signaling system, and focal adhesion (Figure 6B, Supplementary material 3).

## Discussion

N6-methyladenosine is the most prevalent internal form of modification in polyadenylated mRNAs and long non-coding RNAs in higher eukaryotes, having been described in yeast, Arabidopsis, fruit flies, and mammals (31, 32). Recent studies have shown that m6A modifications to mRNA have a variety of biological functions and play key roles in gene expression regulation (33), animal development (16), and human diseases (34).

We aimed to describe the m6A modification profiles in embryonic breast muscle of ducks so as to lay a foundation for further exploring how m6A modifications contribute to the growth and development of duck breast muscle. In addition, we also did miRNAs-seq. MiRNA is also an important regulator of breast muscle development. The combination of the m6A-seq and miRNAs-seq will help us find the key target of breast muscle development.

Skeletal muscle development is a complex and multi-stage process that includes the formation of muscle cells into myotubes and the formation of muscle fibers (35, 36). However, almost no animals increase the number of muscle fibers after birth; thus, the amount of muscle meat production in adult livestock and poultry is determined during embryogenesis. Therefore, it is important to study embryonic muscle development (20).

Gu et al. (20) reported that E19 is the fastest point for breast muscle development. Before the E19 stage, breast muscle is mainly involved in muscle fiber proliferation events, while afterward, the crucial event is muscle fiber fusion to form more multinucleated myotubes. Therefore, we selected breast muscles at the E13, E19, and E27 stages for the preliminary experiment and found that the expression levels of m6A and methylation modification enzymes were all highest at E19. This is consistent with the duck breast muscle development model and suggests that m6A methylation modification plays some key role in the development of embryonic breast muscle of ducks. These results encouraged us to perform m6A sequencing of embryonic breast muscle at the E19 and E27 stages.

Through m6A-seq, we obtained a list of m6A peaks that overlapped with genes at the E19 (7,438) and E27 (7,753) stages, indicating that m6A modification might be a common approach for duck gene regulation. The methylated genes obtained in this study are much higher than those detected in pigs and chickens, respectively, finding 5,864 and 3,303 methylated genes for muscle tissues and adipose tissues of pigs, and 2,893 and 4,593 transcripts for pre- and post-selection follicles (19, 37).

Recently, two independent studies combining m6A immunoprecipitation with high-throughput analysis revealed that the m6A modification tends to occur in the termination codon, 3′UTRs, mRNA exons, and protein-coding regions (12, 38). Our research also manifested similar distribution patterns in which the peaks from m6A-IP samples were highly accumulated around the other exons (except the first exon) in two embryonic periods, accounting for 53.81 and 36.17%, respectively (Figures 3B,C). At the E19 stages, peaks on the first exon and 3′UTR accounted for 18.81 and 18.69%, respectively, while peaks on the first exon and the other exons both accounted for ~36%, suggesting the conservation of m6A distribution in various species.

The skeletal muscle development and fat deposition are both complicated multi-step processes involving some crucial signaling pathways, e.g., initiation and mediation. For skeletal muscle development (39), the Wnt signaling pathway (40), the activation of MAPK activity (41), and the calcium signaling pathway (42) are all tightly associated with skeletal muscle development. For fat deposition, both fatty acid metabolism and peroxisome (43) play key roles. In this study, many MMGs were enriched in both muscle-related pathways (Wnt signaling pathway, calcium signaling pathway, and/or MAPK activity) and fat-related pathways (peroxisome). m6A modification was tightly related to biological processes, including skeletal development and fat deposition, which suggested m6A modification might play crucial roles in duck breast muscle development and fat deposition. Moreover, a negative correlation between m6A methylation enrichment and gene expression levels was found in chicken follicles (37). In addition, our previous study also indicated that E19 was the fastest point of breast muscle development (20). Therefore, we propose that MMGs with lower m6A levels may be the positive regulators for the breast muscle development of ducks, while MMGs with higher m6A levels might be the negative regulators. However, the results obtained above need to be validated in future by molecular experiments.

N6-methyladenosine modification has a regulatory effect on the mRNA of the gene, thus affecting the gene's function. “Writers” were responsible for determining the sex of Drosophila development by adding m6A modifications to the pre-mRNA of Sxl (44). YTHDF2 can regulate the mRNA level during the maternal-to-zygotic transition (MZT) and regulate the development of zebrafish offspring (45). Therefore, association analysis of MMGs and DEGs is important in investigating the regulatory roles of m6A.

In this study, we overlapped MMGs and DEGs and obtained the shared genes. Thus, the shared genes might be the genes affected by m6A. We further picked out 11 muscle-related development genes from the shared genes, including four “up–up” genes (*BCL9, SOX11, EPHB1*, and *MYOCD*) and seven “down–down” genes (*BVES, SLC8A3, ASB2, CFLAR, EPHB1, Wnt1a*, and *SCN4A*) (Table 3), which can be used to explore the regulation mechanism of m6A modification in embryonic breast muscle of ducks. In particular, *Wnt7a* is implicated in playing roles in homeostasis maintenance of skeletal muscle, and *Wnt7a* treatment may be potentially applied in skeletal muscle dystrophy (46). In addition, *Wnt7a* induces satellite cell expansion, myofiber hyperplasia, and hypertrophy in rat craniofacial muscle (47). Therefore, we selected *Wnt7a* for visualization analysis.

It has been reported that the transcript of argonaute-2 (AGO2), a catalytic protein in miRNA-mediated gene silencing, was highly methylated in young peripheral blood mononuclear cells although less so following aging (48). The study also showed a correlation of m6A-methylated AGO2 mRNA with miRNA expression, and it indicated a negative effect of m6A methylation on miRNA expressions during cellular senescence. In this study, we found that six DEMs with their target genes overlapped with genes shared by DEGs and DMGs, suggesting that these six DEMs are regulated by m6A modification. The GO and KEGG analyses for the targets of the six DEMs showed many significantly enriched GO terms or KEGG pathways involved in the regulation of skeletal muscle development. Among them, phosphatidylinositol 3-kinase complex and phosphatidylinositol 3-kinase complex class IA may regulate AMPK activity (49) and subsequently promote skeletal muscle regeneration (50). In addition, phosphatidylinositol phosphorylation, phosphatidylinositol-mediated signaling, and inositol phosphate metabolism are all related to the phosphatidylinositol signaling system. Safi et al. (51) showed that the PI3K pathway, a pathway belonging to the phosphatidylinositol signaling system, can impede the effect of CKIP-1 on C2C12 cell differentiation. Based on our results and the available literature data, we speculate that m6A modification might play a key role in the skeletal muscle development of ducks by affecting miRNA.

In conclusion, we compared the homologous methylase sequences between ducks and humans and illustrated the overall m6A level and the expression of methylases at the E19 and E27 stages. We also revealed the global m6A modification patterns in duck embryonic breast muscles and found that MMGs were enriched in skeletal muscle development-related pathways. In addition, our results strongly indicated that genes shared by DEGs and MMGs might be associated with skeletal muscle development and fat deposition. Finally, we found miRNAs were also regulated by m6A modification, revealed by association analysis of miRNA-seq, RNA-seq, and m6A-seq data.

## Data availability statement

The datasets presented in this study can be found in online repositories. The names of the repository/repositories and accession number(s) can be found in the article/Supplementary material.

## Ethics statement

The animal study was reviewed and approved by Animal Care and Use of Chinese Academy of Tropical Agricultural Sciences.

## Author contributions

LG and TX prepared the manuscript and designed the experiments. LG, SZ, BL, QJ, TX, YH, DL, MX, LH, FW, XZ, ZC, and WS collected the samples. TX was responsible for the design and direction of the experiment. All authors have read and agreed to the published version of the manuscript.

## Funding

This work was supported by the National Natural Science Foundation of China General Program (31972553), Key Research and Development Programs of Hainan Province (ZDYF2022XDNY154), Central Public-interest Scientific Institution Basal Research Fund for Chinese Academy of Tropical Agricultural Sciences (630032017034 and 1630032022013), Chinese Modern Technology System of Agricultural Industry (CARS-42-50), and Special Funds for Central Government Guiding Local Science and Technology Development (ZY2019HN01).

## Conflict of interest

The authors declare that the research was conducted in the absence of any commercial or financial relationships that could be construed as a potential conflict of interest. The reviewer SR declared a shared affiliation with the authors YH and SZ to the handling editor at the time of review.

## Publisher's note

All claims expressed in this article are solely those of the authors and do not necessarily represent those of their affiliated organizations, or those of the publisher, the editors and the reviewers. Any product that may be evaluated in this article, or claim that may be made by its manufacturer, is not guaranteed or endorsed by the publisher.
