# Supplementary material for: m6A and miRNA jointly regulate the development of breast muscles in duck embryonic stages
Source: Front Vet Sci. 2022 Oct 24;9:933850. doi: 10.3389/fvets.2022.933850 (PMC9637736; doi:10.3389/fvets.2022.933850)
Supplement: Supplementary file 4 [file Table_4.DOC]

Table S1. Basic information of sequenced data

| Sample | Clean reads | Mapped reads | Mapped % | Unique-mapped reads | Unique-mapped % | Multi-mapped reads | Multi-mapped % |
| --- | --- | --- | --- | --- | --- | --- | --- |
| RL15_1_IP | 71000822 | 53938184 | 75.97 | 41975686 | 59.12 | 11962498 | 16.85 |
| RL15_2_IP | 67852604 | 51955120 | 76.57 | 41620249 | 61.34 | 10334871 | 15.23 |
| RL15_3_IP | 61802672 | 47594650 | 77.01 | 37539551 | 60.74 | 10055099 | 16.27 |
| RL27_1_IP | 57826938 | 43982314 | 76.06 | 33968048 | 58.74 | 10014266 | 17.32 |
| RL27_2_IP | 54903112 | 42181909 | 76.83 | 33276833 | 60.61 | 8905076 | 16.22 |
| RL27_3_IP | 56294966 | 42363693 | 75.25 | 32804753 | 58.27 | 9558940 | 16.98 |
| RL15_1_input | 58424614 | 44114452 | 75.51 | 36307194 | 62.14 | 7807258 | 13.36 |
| RL15_2_input | 71608208 | 55199618 | 77.09 | 45986975 | 64.22 | 9212643 | 12.87 |
| RL15_3_input | 59703012 | 46339719 | 77.62 | 37279827 | 62.44 | 9059892 | 15.17 |
| RL27_1_input | 71885174 | 55727154 | 77.52 | 45538908 | 63.35 | 10188246 | 14.17 |
| RL27_2_input | 72331246 | 55603772 | 76.87 | 45010476 | 62.23 | 10593296 | 14.65 |
| RL27_3_input | 72995406 | 54346382 | 74.45 | 44107923 | 60.43 | 10238459 | 14.03 |
